# Removing the Threat of Diclofenac to Critically Endangered Asian Vultures

**DOI:** 10.1371/journal.pbio.0040066

**Published:** 2006-01-31

**Authors:** Gerry Swan, Vinasan Naidoo, Richard Cuthbert, Rhys E Green, Deborah J Pain, Devendra Swarup, Vibhu Prakash, Mark Taggart, Lizette Bekker, Devojit Das, Jörg Diekmann, Maria Diekmann, Elmarié Killian, Andy Meharg, Ramesh Chandra Patra, Mohini Saini, Kerri Wolter

**Affiliations:** **1**Department of Paraclinical Sciences, and Biomedical Research Centre, Faculty of Veterinary Science, University of Pretoria, Onderstepoort, South Africa; **2**Royal Society for the Protection of Birds, The Lodge, Sandy, Bedfordshire, United Kingdom; **3**Conservation Biology Group, Department of Zoology, University of Cambridge, Cambridge, United Kingdom; **4**Indian Veterinary Research Institute, Izatnagar, Uttar Pradesh, India; **5**Bombay Natural History Society, Mumbai, India; **6**School of Biological Sciences, Department of Plant and Soil Science, University of Aberdeen, Aberdeen, United Kingdom; **7**Rare and Endangered Species Trust, Otjiwarongo, Namibia; **8**Vulture Unit, DeWildt Cheetah and Wildlife Trust, Hartbeespoort, South Africa; Institute of ZoologyUnited Kingdom

## Abstract

Veterinary use of the nonsteroidal anti-inflammatory (NSAID) drug diclofenac in South Asia has resulted in the collapse of populations of three vulture species of the genus*Gyps* to the most severe category of global extinction risk. Vultures are exposed to diclofenac when scavenging on livestock treated with the drug shortly before death. Diclofenac causes kidney damage, increased serum uric acid concentrations, visceral gout, and death. Concern about this issue led the Indian Government to announce its intention to ban the veterinary use of diclofenac by September 2005. Implementation of a ban is still in progress late in 2005, and to facilitate this we sought potential alternative NSAIDs by obtaining information from captive bird collections worldwide. We found that the NSAID meloxicam had been administered to 35 captive*Gyps* vultures with no apparent ill effects. We then undertook a phased programme of safety testing of meloxicam on the African white-backed vulture*Gyps africanus,* which we had previously established to be as susceptible to diclofenac poisoning as the endangered Asian*Gyps* vultures. We estimated the likely maximum level of exposure (MLE) of wild vultures and dosed birds by gavage (oral administration) with increasing quantities of the drug until the likely MLE was exceeded in a sample of 40G. africanus. Subsequently, sixG. africanus were fed tissues from cattle which had been treated with a higher than standard veterinary course of meloxicam prior to death. In the final phase, ten Asian vultures of two of the endangered species*(Gyps bengalensis*,*Gyps indicus)* were dosed with meloxicam by gavage; five of them at more than the likely MLE dosage. All meloxicam-treated birds survived all treatments, and none suffered any obvious clinical effects. Serum uric acid concentrations remained within the normal limits throughout, and were significantly lower than those from birds treated with diclofenac in other studies. We conclude that meloxicam is of low toxicity to*Gyps* vultures and that its use in place of diclofenac would reduce vulture mortality substantially in the Indian subcontinent. Meloxicam is already available for veterinary use in India.

## Abstract 2

Phased testing reveals that the anti-inflammatory drug meloxicam is less toxic to endangered*Gyps* vultures than diclofenac and, if used as an alternative, could potentially reverse the catastrophic decline of these species in South Asia.

## Introduction

Veterinary use of the nonsteroidal anti-inflammatory drug (NSAID) diclofenac is a major cause of the catastrophic collapse of*Gyps* vulture populations in the Indian subcontinent [1–3]. Three species of vultures endemic to South Asia, which together used to number tens of millions, are now at high risk of global extinction and are listed as critically endangered [4]. Populations of Oriental white-backed*(Gyps bengalensis),* long-billed*(Gyps indicus)* and slender-billed vultures*(Gyps tenuirostris)* have declined by more than 95% since the early 1990s [5,6], and continue to decline at an annual rate of 22% to 48% [3].

Diclofenac is a widely available veterinary drug in the Indian subcontinent, where it is used for the symptomatic treatment and management of inflammation, fever, and/or pain associated with disease or injury in domestic livestock. Vultures are exposed to the drug when they consume carcasses of cattle that were treated with diclofenac shortly before death. Following experimental exposure to diclofenac or diclofenac-contaminated tissues,*Gyps* vultures die within days from kidney failure with clinical signs of extensive visceral gout (formation of uric acid crystals within tissue) [1,7]. These clinical signs and diclofenac residues in vulture tissues have been found in carcasses of wild*Gyps* vultures from across India, Pakistan, and Nepal [1,2], and the proportion of vulture carcasses with signs of diclofenac poisoning is consistent with this being the main, and possibly the only, cause of the vulture decline [3].

The loss of tens of millions of vultures over the last decade has had major ecological consequences across the Indian subcontinent that pose a potential threat to human health. In many places, populations of feral dogs*(Canis familiaris)* have benefited from the disappearance of*Gyps* vultures as the main scavenger of wild and domestic ungulate carcasses [8]. Associated with the rise in dog numbers [9] is an increased risk of human cases of rabies. If rat (*Rattus* spp.) populations also increase at carcass dumps in and near settlements, the risk of transmission of diseases, including bubonic plague, to humans may also increase. Vultures probably also helped to control livestock diseases, such as brucellosis, tuberculosis, and anthrax by disposing of infected carcasses [10,11]. The loss of vultures has had a social impact on the Indian Zoroastrian Parsi community, who traditionally use vultures to dispose of human corpses in “sky burials” [12] and are now having to seek alternative disposal methods [13]. As a consequence of the collapse of vulture populations, national and international conservation organisations have concluded that it is essential to ban the use of diclofenac in livestock so as to remove it as a contaminant of the food of wild vultures [14]. At a meeting of the National Wildlife Board in March 2005, the Government of India announced that it intended to phase out the veterinary use of diclofenac [15].

The identification of NSAIDs that are effective for the treatment of livestock, but also relatively nontoxic to vultures, would facilitate the removal of diclofenac from the food of vultures. NSAIDs are characterised by their ability to inhibit cyclo-oxygenase enzymes, which are involved in the formation of prostaglandins. However, there are marked differences between drugs in their selective inhibition of the two subtypes of cyclo-oxygenase COX-1 and COX-2, with the latter being involved with the modulation of inflammatory responses and pain, while the former modulates blood flow to the kidneys. The ability of NSAIDs to inhibit both these subtypes has been implicated as a cause of the severe side effects occasionally associated with the use of some NSAIDs [16]. Toxic effects on the kidneys of birds have been observed following treatment with a number of NSAIDs [1,17]. However, there are marked interspecific differences in toxicity [18–20], and it is necessary to establish the safety of individual NSAIDs to*Gyps* vultures. To identify candidate alternative drugs, we contacted veterinarians at zoos and wildlife rehabilitation centres worldwide and requested information on the clinical use of NSAIDs on captive*Gyps* vultures, including the outcome of such treatment. Preliminary results suggested that the NSAID meloxicam is a potential alternative for diclofenac, because 35 individuals from six*Gyps* species (including five Oriental white-backed vultures) treated with meloxicam, typically at doses of 0.2–0.5 mg kg^−1^, showed no ill effects; while the use of several other NSAIDs was associated with renal failure (unpublished data).

As all three of the resident Asian*Gyps* vultures are critically endangered, we considered it unacceptable to use these species for safety testing without first evaluating the safety of meloxicam on a suitable surrogate. The African white-backed vulture*(Gyps africanus)* was chosen as a surrogate because it has a favourable global conservation status (category Least Concern) [4], and diclofenac has been shown experimentally to be as toxic to it as it is to the endangeredG. bengalensis [7]. Clinical signs at postmortem examination of experimentally dosed birds indicate a similar mechanism of toxicity in both species. Diclofenac-dosedG. africanus showed significant increases in serum uric acid concentrations 12–24 h after dosing and exhibited lethargy and neck-drooping behaviour before death [7].

In this paper, we report tests on the safety of meloxicam to*Gyps* vultures, which we dosed with meloxicam by gavage (oral administration) and by feeding them with tissue from meloxicam-dosed cattle. Using both routes of drug administration, the range of dose levels exceeded our estimated likely maximum level of exposure (MLE) of meloxicam to wild vultures. To minimise the risk of suffering and death of experimental animals, safety testing was undertaken in six phases (summarised inTable 1). During the first three phases, the dose rate of meloxicam administered by gavage toG. africanus was progressively increased from 0.5 mg kg^−1^ vulture body weight to 1 mg kg^−1^ and then to the highest dose of 2 mg kg^−1^, which exceeds our estimate of the MLE (Protocol S1). At the conclusion of each phase the results were evaluated, and the study only proceeded to the next phase if all of the dosed birds were healthy and had clinically normal serum concentrations of uric acid and alanine transferase (ALT), both of which are known to be elevated beyond the normal range inG. africanus after treatment with diclofenac [7]. In the fourth phase, meloxicam was administered at 2 mg kg^−1^ to captive*G. africanus* in South Africa and wild vultures in Namibia, thereby exposing a larger number of vultures from two distinct populations to the estimated MLE of meloxicam in the wild. The fifth phase of the study simulated the natural route of NSAID exposure, by feeding vultures with liver and muscle tissue from cattle that had received a higher than standard veterinary course of meloxicam treatment, with daily injections over five days. The final phase of testing was to assess the safety of meloxicam to two of the three critically endangered Asian vultures, by administering meloxicam by gavage to captiveG. bengalensis andG. indicus in India.

**Table 1:**
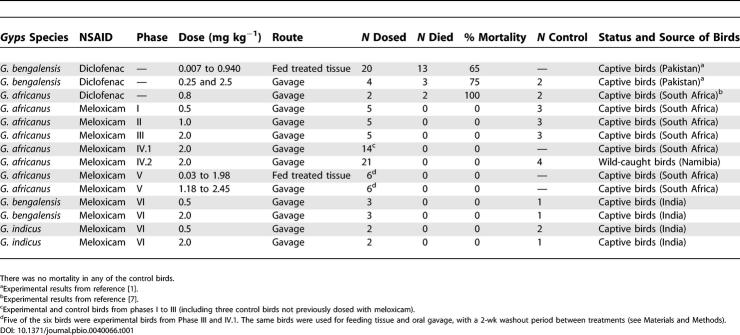
Summary of Results and Experimental Schedule for the Testing of the NSAIDs Diclofenac and Meloxicam onG. bengalensis andG. indicus Vultures, and on the NonthreatenedG. africanus

## Results and Discussion

### Safety Testing Using CaptiveG. africanus

In each of the first three phases of our study, we administered a single dose of meloxicam to five vultures by gavage (oral administration into the crop via a 5-mm tube) and gave sterilised water to three control birds by the same method. The birds' apparent health and serum parameters were then assessed for 7 d after treatment. Dose rates in Phases I to III were 0.5, 1, or 2 mg kg^−1^, respectively, and were set so that the highest dose just exceeded the likely MLE of wild vultures (estimated as 1.83 mg kg^−1^ vulture body weight;Protocol S1). No ill health was observed in any of the 15 vultures treated with meloxicam at these three dose levels, and all birds were alive and healthy at the end of the experimental period (Table 1). There was a significant loss of body mass during the experimental period in Phases I, II, and III (matched pairs*t* test; Phase I*t*_7_ = 7.28,*p* < 0.001; Phase II*t*_7_ = 2.97,*p* < 0.05; Phase III*t*_7_ = 2.96,*p* < 0.05). However, there was no significant difference between the meloxicam-dosed and control birds in body mass change as a percentage of initial mass in any of the three Phases (2-sample*t* test; Phase I*t*_6_ = 0.13,*p* > 0.89; Phase II*t*_6_ = 0.46,*p* > 0.66; Phase III*t*_6_ = 0.61,*p* > 0.56). Because of this, and because no significant loss of body mass was observed in later phases of the experiment, when birds were handled for sampling on fewer occasions and not moved from their normal holding aviaries (see below), we believe that the loss of body mass was most likely due to the stress caused by handling and sampling, rather than to meloxicam.

We compared the survival of vultures in these experiments with that of twoG. africanus treated with comparable doses of diclofenac using the same methods [7]. In each phase, all five meloxicam-treated vultures survived the experimental period, whereas both diclofenac-treated birds died with extensive visceral gout. This represents a statistically significant difference in death rate between the two drugs (2-tailed Fisher exact test; 0/5 deaths versus 2/2 deaths,*p* = 0.0476 in each phase). However, because of the small sample sizes, these results do not exclude the possibility that, in a worst-case scenario, meloxicam might have caused appreciable mortality if used on a larger sample. For example, with a total sample of 15 treated birds, statistically there could still be a 5% chance of no birds dying, even if the true probability of death per trial was as high as 18% ((1–0.18)^15^ = 0.05). If only the five birds treated in Phase III with more than the MLE are considered, the failure to observe any deaths implies that there could be a 5% probability that the true risk of death per trial might be as high as 45% ((1–0.45)^5^ = 0.05), which led us to test a larger sample of birds in Phases IV and V (see below).

**Figure 1:**
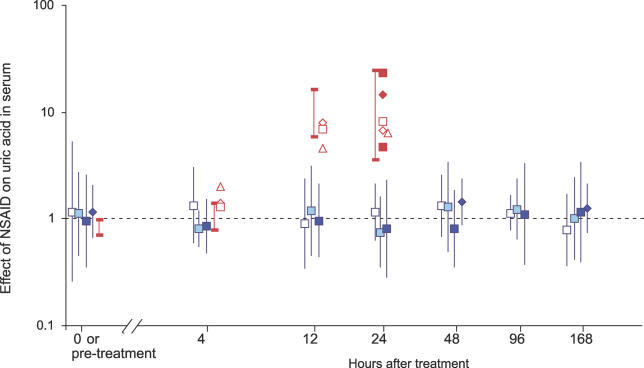
Effect of Administration of Meloxicam and Diclofenac by Gavage on Uric Acid in the Serum of Vultures Blue symbols show the ratio of the geometric mean serum concentration of uric acid for a group ofGyps africanus treated with meloxicam by gavage to that for a control group treated with water and sampled at the same time. Vertical lines show 95% confidence limits for the ratio. The dashed horizontal line indicates a ratio of 1; i.e., no effect of treatment. For each of six samplings after treatment, results are shown for experiments in which different doses of drug were used. The fill colour of the blue symbols indicates the meloxicam dose for the treated group: white = 0.5 mg kg^−1^ (Phase I); light blue = 1.0 mg kg^−1^ (Phase II); dark blue = 2.0 mg kg^−1^ (squares = Phase III, diamonds = Phase IV-2). Red vertical bars show the maximum and minimum values of the equivalent ratio for two groups of*G. africanus,* one group treated with 0.8 mg kg^−1^ of diclofenac by gavage and another group treated with water and sampled at the same time. Open red symbols show the ratio of the serum concentration after treatment to that at the time of treatment for three individualG. fulvus given 0.8 mg kg^−1^ of diclofenac by gavage. Filled red symbols show the ratio of the serum concentration 24 h post-treatment to that 1 h post-treatment for three individualG. bengalensis given 0.25 mg kg^−1^ (squares) and 2.5 mg kg^−1^ (diamond) of diclofenac by gavage. Data from diclofenac experiments were taken from references [1] and [7].

Although the survival of all of the meloxicam-treated vultures in Phases I–III is not robust evidence of safety on its own, it can be combined with information obtained by sampling the blood of experimental and control birds. There were no significant differences in serum concentrations of uric acid, ALT, albumin, and creatinine kinase (CK) between treated and control groups in any of the three phases and for any of the sampling times after dosing (Table S1). Inspection of the magnitude of average differences in serum concentrations between treated and untreated birds showed no indication of a systematic trend for any of the serum constituents in relation to dose (Figure 1,Table S1). Since the serum concentration of uric acid has been shown to be elevated well beyond the normal range in*G. africanus, G.bengalensis,* andGyps fulvus treated with comparable fatal doses of diclofenac [1,7], these observations provide substantial further evidence of safety.

### Safety Testing Using Larger Numbers of Captive and Wild-CaughtG. africanus

Our objective in the next phase of the study was to narrow the range of possible values of the true rate of meloxicam-induced mortality that would be consistent with our data by testing larger numbers of vultures with more than the likely MLE. In this phase, we treated two groups ofG. africanus. In Phase IV.1, we used 14 long-term captive birds that had been used more than 6 wk previously in Phases I to III (11 as experimental birds and three as controls). We treated all 14 birds with meloxicam. In Phase IV.2, we captured 25 wildG. africanus in Namibia and held them temporarily. Of these birds, 21 were treated with meloxicam and four received sterilised water and acted as controls. All treated birds in Phase IV were given 2 mg kg^−1^ of meloxicam by gavage (Table 1).

All 35 meloxicam-treated birds survived the 7-d experimental period, and the wild-caught vultures used in Phase IV.2 were all successfully released after the experiment. There was no significant change in the body mass of meloxicam-treated birds between the beginning and end of the 7-d period for either captive (matched pairs*t* test;*t*_13_ = 0.29,*p* > 0.77) or wild-caught birds (matched pairs*t* test;*t*_24_ = 1.68,*p* > 0.10). For the wild-caught birds there was also no significant difference in the percentage mass change of meloxicam-dosed and control birds (2-sample*t* test; Phase I*t*_23_ = 0.30,*p >* 0.77). Serum uric acid concentrations did not differ significantly between experimental and control groups and showed no trend during the experimental period (Figure 1,Table S1). Neck-drooping behaviour, similar to that seen in diclofenac-dosed birds [7], was observed in the Phase IV.2 birds soon after the collection of the second blood sample at 48 h following treatment, and two birds lay on the ground. However, neck-drooping was observed in both meloxicam-dosed and control birds, and occurred during the heat of the day. Hence, we consider that the neck-drooping we observed was most likely to be a thermoregulatory activity [21] in response to high ambient temperature and an elevation of body temperature caused by the stress of handling and sampling, rather than a response to meloxicam treatment. By the end of the day, all birds (including the two recumbent birds) had resumed a normal body posture. Neck-drooping was not observed over the remaining 5 d of the trial. Hence, we consider it to be a nonspecific response to stress caused by heat or handling and not a specific response to NSAID poisoning.

When the results from Phases III and IV of the study are combined, 40G. africanus were treated by gavage with more than the likely MLE of meloxicam, and all survived with no ill effects observed that were attributable to the drug. These data indicate a 95% probability that the true probability of death per trial consistent with these data was no higher than 7% ((1−0.07)^40^ = 0.05). Taken together with the evidence of lack of an effect of meloxicam on serum uric acid concentrations, these results indicate that meloxicam administered by gavage does not cause appreciable mortality inG. africanus.

### Safety Testing by FeedingG. africanus on Tissues of Meloxicam-Treated Cattle

We wished to assess the possibility that although meloxicam itself appears safe when administered to vultures at the MLE, metabolites produced by treated cattle might be toxic. To test this, we gave daily injections of 1.0 mg kg^−1^ of meloxicam to three cattle*(Bos taurus)* for 5 d. This is a higher dose level than the two standard veterinary doses recommended in India (0.5 to 0.7 mg kg^−1^ daily for five consecutive days). We slaughtered the three cattle 8 h after the last injection and fed liver or muscle to six captive*G. africanus*. An experiment by the European Agency for the Evaluation of Medicinal Products (EMEA) onBos taurus found that tissue meloxicam concentrations in treated animals were higher in liver than in other tissues tested, and peaked at the 8-h sampling period (Protocol S1) [20]. In our experiment, concentrations of parent meloxicam in cattle tissues at slaughter averaged 0.50 ± 0.13 (± 1 standard deviation) mg kg^−1^ for muscle and 8.12 ± 1.10 mg kg^−1^ for liver. Vultures consumed an average of 0.59 ± 0.21 (± 1 standard deviation) kg of liver and 0.67 ± 0.32 kg of muscle tissue, of the 1 kg with which they were each provided, within the 48-h feeding period. On one occasion, a bird ate all of the liver provided, and on two occasions, birds ate the entire portion of muscle. The dose of parent meloxicam ingested ranged from 0.03–0.15 mg kg^−1^ vulture body weight for muscle, and from 0.57–1.98 mg kg^−1^ body weight for birds feeding on liver.

Because we administered meloxicam for 5 d at a higher dose (1.0 mg kg^−1^) than in the EMEA study (0.7 mg kg^−1^) [22], the maximum dose ingested by a vulture (1.98 mg kg^−1^ bw) and the maximum cattle liver tissue concentrations (8.91 mg kg^−1^) are somewhat higher than those predicted from the EMEA work (Protocol S1). For comparison, we also administered meloxicam by gavage at doses (1.18–2.45 mg kg^−1^ vulture body weight) intended to be similar to those ingested by birds feeding on liver. All six birds survived the treatments and no ill effects or altered feeding behaviour was observed. There was no significant change in body mass between the start and end of the 5-d experimental period for any of the three treatment types (matched pairs*t* test; muscle*t*_5_ = 1.00,*p* > 0.36; liver*t*_5_ = 2.44,*p* > 0.05; gavage*t*_5_ = 1.46,*p* > 0.20).

**Figure 2:**
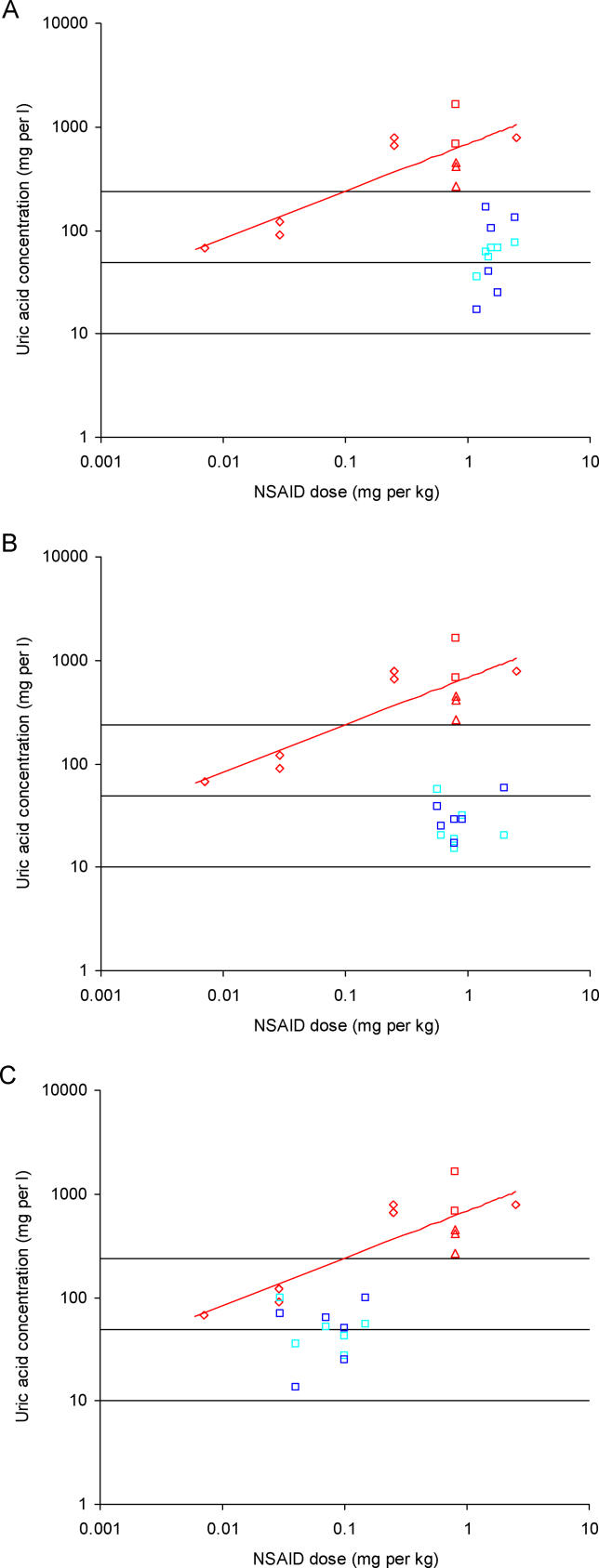
Relationship of Uric Acid in Serum to the Dose of Meloxicam and Diclofenac Administered and to the Administration Method Serum concentration of uric acid inGyps africanus 48 h (turquoise) and 96 h (blue) after treatment, in relation to the dose of meloxicam administered per kg of vulture body weight. For comparison, the geometric mean uric acid level (central horizontal line) and 95% range (upper and lower horizontal lines) of the experimental birds 24 h before treatment are shown. Also shown are serum concentrations of uric acid 24 h after treatment inG. africanus (red squares),G. bengalensis (red diamonds), andG. fulvus (red triangles), to which diclofenac was administered by various methods. The red line shows the regression model fitted to these data. Panels show results for different methods of administration of meloxicam to*G. africanus:* (A) gavage, (B) by feeding liver from meloxicam treated cattle, (C) by feeding muscle from meloxicam-treated cattle. Data from diclofenac experiments were taken from references [1] and [7].

Serum uric acid concentrations remained within the 95% range observed in these individuals before treatment at both sampling times and also within the similar 95% ranges for uric acid for wildG. africanus captured in Namibia and reported forG. africanus captured in Kenya [23] (Figure 2). There was no significant relationship between uric acid concentration and meloxicam dose at 48 h or 96 h (OLS regressions of log uric acid concentration on log meloxicam dose for each of the three administration routes;*p* > 0.05 in all cases). This was also the case when the log of the ratio of the uric acid concentration after treatment to that before treatment was used as the dependent variable. A more elaborate analysis of variance in which log uric acid concentration was modelled as a function of treatment method, time period, and log meloxicam dose, with pretreatment log uric acid concentration as a covariate, also gave no indication of any significant effect on serum uric concentration of treatment with meloxicam by any of the three routes (Protocol S2). The absence of mortality or elevation of serum uric acid levels indicates that tissues of cattle treated with meloxicam shortly before death are unlikely to be toxic toG. africanus. The experiments using liver tissue are particularly informative, because the quantity of liver eaten by one bird approached the maximum meal size likely to be consumed by a wild vulture, and this bird received a dose of parent meloxicam in excess of the likely MLE.

### Safety Testing of Meloxicam on Endangered Asian*Gyps*

Although the experiments we have reported so far indicate that meloxicam appears safe for*G. africanus,* this does not exclude the possibility that it might be toxic to Asian*Gyps* species, though this seems unlikely in view of the close phylogenetic relationships within the genus [24] and the similarity of the response to diclofenac ofG. africanus andG. bengalensis. We therefore administered meloxicam doses of 0.5 mg kg^−1^ by gavage to three captiveG. bengalensis and twoG. indicus and the MLE of 2.0 mg kg^−1^ to threeG. bengalensis and twoG. indicus. All 10 meloxicam-treated birds survived the 7-d experimental period and they remain alive and healthy 4 mo afterwards. None showed signs of ill health or abnormal behaviour. There was no significant change in body mass during the experimental period (paired t-test;*t*_5_ = 2.07,*p* > 0.09).

Hence, although the number of birds tested was small, there is no indication of adverse effects of meloxicam on these two species of Asian*Gyps* vultures.

### Conclusions

The results of this study demonstrate that meloxicam is much less toxic than diclofenac in at least three*Gyps* species, including two of the critically endangered Asian species. Indeed, we found no evidence that meloxicam administered at doses exceeding our estimated likely MLE caused any deaths or even elevation of serum uric acid concentrations. Combining the results of this study with those from the questionnaire to zoo veterinarians, a total of at least 88 individual birds from seven*Gyps* species are known to have received meloxicam at various doses with no recognized adverse effects. Hence, with this total of treated birds there is a 95% chance that the per trial probability of mortality caused by meloxicam is no higher than 3.5%. The observation that serum concentrations of uric acid remain within the normal range for all meloxicam dose rates adds substantially to the evidence that meloxicam has low toxicity to*G. africanus,* given that uric acid concentrations in this and two other*Gyps* species were markedly elevated by lethal treatment with diclofenac [1,7]. Preliminary results from the NSAID questionnaires indicate the safety of meloxicam to a wide range of other vultures, raptors, and scavenging bird species, and to date we know of more than 700 individuals from more than 30 species that have been treated with no apparent adverse effects (unpublished data). This demonstrates that, at recommended clinical dose levels, meloxicam is not toxic to a wide range of avian species.

Any replacement for diclofenac must be effective for the treatment of livestock as well as safe for vultures. Meloxicam is one of the newer NSAIDs with preferential COX-2 inhibition, having analgesic, antipyretic, and anti-inflammatory properties and a reduced risk of adverse effect on renal function [16,25]. It is used to treat a variety of veterinary ailments [26–30], and it is rated as a highly effective NSAID [30–32]. Meloxicam is approved for human use in more than 80 countries, including India [33,34]. It is used and licensed as a veterinary drug in India, Europe, and North America [35,36] and is already manufactured in India, where, like diclofenac, it is available as both an injectable solution and oral bolus. We hope that efforts to prevent diclofenac being used to treat domestic livestock in the Indian subcontinent and in other*Gyps* vulture range states will continue as a matter of urgency. Where the availability of alternative drugs is seen as a barrier to achieving this objective, we recommend that governments consider advocating the use of meloxicam as an alternative to diclofenac. Because vulture populations are now very low and contamination of even a small proportion of livestock carcasses is sufficient to cause adverse impacts on vulture populations [3], we also advocate immediate intensification of efforts to establish viable captive populations of all three critically endangered species.

## Materials and Methods

### Trial animals

Nonreleasable captive vultures held at the de Wildt Cheetah and Wildlife Trust (South Africa) were used for Phases I–III, Phase IV.1, and Phase V. All birds at de Wildt were habituated to captivity and eating regularly. In Phase IV.2, wildG. africanus (*n =* 25) were captured using a walk-in trap located at a feeding site for vultures in Namibia [37], run by the Rare and Endangered Species Trust. CaptiveG. bengalensis andG. indicus for Phase VI of the trials were held at the Bombay Natural History Society/Haryana State Vulture Conservation Breeding Centre, Pinjore, Haryana State, India. All birds used in Phase I–VI were adults and subadults. Ethical issues relating to the experimental protocols were considered and approved by the Animal Use and Care Committee and the Research Committee of the Faculty of Veterinary Science of the University of Pretoria, the Research Council of the Indian Veterinary Research Institute, and the Board of the Bombay Natural History Society.

### Housing and management

Birds used for Phases I–III were transported from De Wildt to the University of Pretoria Biomedical Research Centre (UPBRC) 7 d prior to the start of Phases I–III. At the UPBRC, vultures were housed individually in primate cages (1.2 × 0.87 × 0.78 m) in an environmentally controlled room in which the temperature (19–22 °C) and light cycle were kept constant and humidity was allowed to vary with that outside (between 19% and 50% humidity). Vultures used for Phase IV.1 and Phase V were kept at De Wildt, either within their normal holding aviaries (IV.1), or within smaller isolation cages (V). Birds captured in Namibia (Phase IV.2) were kept in the walk-in trap (11 × 5.5 × 5.5 m) [37], which doubled as a holding aviary for the 7-d trial. Birds in India were captured from their flight aviaries 6 d before the start of the trials. Five birds with pre-existing healed wing or leg injuries were held in three small aviaries (4 × 3 × 2.5 m), the remaining two groups of five birds were kept in two large holding aviaries (15 × 10 × 5 m). The vultures were not fed for 24 h prior to treatment with meloxicam and for up to 4 h afterwards. Thereafter, birds were fed according to their normal feeding regime (200 g of meat daily at De Wildt and 1.0 kg of meat every third or fourth day at Pinjore), with the exception of the wild birds in Namibia, which were free to feed from the remains of an adult donkey*(Equus asinus)* placed in the aviary. All meat was from known sources, which were selected because we were confident that they did not use any NSAIDs on their livestock.

### Treatment and study design for oral gavage experiments

Phases I–III followed a randomised, two-treatment–group, parallel-study design with 24 nonreleasable captiveG. africanus. In each phase (I–III), vultures were randomly allocated to a meloxicam-treated group (*n* = 5) and a control group (*n* = 3). In Phase IV.1, we treated 14 captive vultures (no controls), and in Phase IV.2, we treated 21 wild vultures and there were four control birds (Table 1). The vultures used in Phase IV.1 had also been used in Phases I–III. To minimise the chance of any effect of earlier treatment we ensured that the interval between the end of one treatment and the beginning of the next was at least 6 wk. To minimise the risk to captiveG. bengalensis andG. indicus in India, Phase VI of the meloxicam testing was staggered. Two injured nonreleasable birds were first treated by gavage with 0.5 mg kg^−1^ and one control bird was sham-dosed with sterilised water. After 48 h no apparent ill effects of the treatment were observed, so a further three birds were dosed with 0.5 mg kg^−1^, two injured nonreleasable birds were dosed with 2 mg kg^−1^, and a further two control birds were sham-dosed. After another 48 h, three more birds were dosed with 2 mg kg^−1^ along with two final control birds. All birds (with the exception of birds fed muscle and liver tissue in Phase V) were administered meloxicam as a single dose by oral gavage, with the gavage tube flushed with 2 ml of water. Control birds were sham-treated by gavage with sterilised water. Birds were observed following dosing for any regurgitation, but none occurred. The meloxicam used came from >20 bottles of the product purchased from several pharmacies in India. Meloxicam used in all phases of the study was Melonex (Intas Pharmaceuticals, Ahmedebad, India). The stated concentration of meloxicam (500 mg l^−1^) within two bottles was verified against pure meloxicam sodium salt (M-3935, Sigma-Aldrich, St. Louis, Missouri, United States) through the high-performance liquid chromatography (HPLC) analysis method described below and found to be within the accepted 10% limits for pharmaceutical products (450 mg l^−1^ and 460 mg l^−1^).

### Phase V treatment and design

Phase V used a randomized, three-period, three-treatment crossover design with a washout period of 2 wk between repeat dosing. Pharamacokinetic studies indicate that meloxicam is rapidly metabolised in five other bird species (elimination half-life [*t*_½ el_] of 0.5–2.4 h [20]) and eliminated within 12 h in*G. africanus,* and the 2-wk washout period was chosen to ensure that no meloxicam residues were likely to be present on repeat dosing. It was intended that each bird should receive all three treatments in turn with a 2-wk washout period between treatments. The three treatments were (1) feeding with muscle from a meloxicam-treated cow, (2) feeding with liver from a meloxicam-treated cow, and (3) oral gavage with a dose of meloxicam intended to be similar to that taken in treatment (2). In each of the three treatment periods, all three treatments were administered to two birds. Hence, two birds were allotted at random to receive the sequence 1,2,3, two to receive 2,3,1, and two to receive 3,1,2. In each treatment period, the muscle and liver was taken from one cow. In practice, an error was made so that two birds received the wrong treatment in the final period and instead received 2,3,2 and 3,1,1. Hence, although all three treatments were each administered on six occasions, and to two birds in each of the three periods, two birds received the same type of treatment in two periods. All six birds had previously been trained to consume food from bowls. On the day of dosing, two birds were presented with 1 kg of muscle, two birds with 1 kg of liver tissue, and two birds were dosed by oral gavage. Any food remaining after 48 h was removed and weighed. Doses of meloxicam per kg vulture body weight were estimated from the mass of tissue consumed and the concentration of meloxicam within cattle tissues (see below). In the first part of this experiment, neither of the two birds given liver ate much of it, so all six birds were routinely fed liver (between testing sessions) to habituate them to eating liver in the trials.

### Treatment of meloxicam-dosed cattle for Phase V

ThreeBos taurus steers of about 18 mo of age and weighing 300–400 kg were housed at the UPBRC. Each animal received an intramuscular injection of meloxicam at a dose of 1 mg kg^−1^ on each of 5 d prior to slaughter. To avoid unnecessary pain, the drug volume injected into any one site never exceeded 20 ml, with all injections placed in the neck on the left and right side on alternating days. This dose is twice the lower of the two standard doses (0.5 and 0.7 mg kg^−1^) recommended for veterinary medicine in India. It is also higher than the dose (0.7 mg kg^−1^) administered in the EMEA study [22] that we used to calculate the likely MLE of vultures to meloxicam in the wild (Protocol S1). Cattle were slaughtered at the Veterinary Pathology Department, University of Pretoria, by means of captive bolt to the brain followed by the transection of the spinal cord at the level of the atlanto-occipital junction, without subsequent exsanguination. Each animal was slaughtered 8 h after the last meloxicam dose and on the day prior to vulture feeding. The entire liver and quadriceps femoris muscle were collected (sufficient to supply liver and muscle for two vultures) and refrigerated until feeding on the following day.

### Measuring meloxicam in tissues

Meloxicam concentrations in liver and muscle tissues were measured through standard HPLC methods calibrated against a known standard concentration of the drug. Two 1-kg pieces of liver and muscle were cut from each slaughtered animal. Five subsamples of tissue weighing 3–5 g (four from the surface and one from the centre) were taken from each 1-kg block and homogenised. Meloxicam was extracted from a 0.5-g sample of the homogenised tissue, through homogenisation with 2 ml of HPLC grade acetonitrile, which was then centrifuged at 1200 rpm for 10 min and subsequently dried at 60 °C under a flow of nitrogen. This was followed up by a cleanup process using Waters Oasis (Milford, Masachusetts, United States) HLB solid-phase extraction cartridges [38]. The dried eluate was reconstituted in 50 μl MeOH and 100 μl 0.4% AcAc in MeOH:MeOH (40:60) and analysed in duplicate by HPLC. For each homogenised sample, the mean of the four values was used as the final estimate of meloxicam concentrations. Meloxicam sodium salt (M-3935, Sigma-Aldrich) was used for calibration, with nine standards ranging from 100 μg to 50,000 μg l^−1^. The HPLC apparatus comprised a model 126 dual solvent pump, model 168 diode array detector, and a 508 autosampler (Beckman Instruments, Fullerton, California, United States). Chromatographic separation was achieved using a Synergi MAX-RP C18 column (2.1 mm × 150 mm, 5 μm; Phenomenex, Torrance, California, United States) with UV detection at 275 nm, e.g., quantification was done with peak areas acquired from UV detection at 275 nm.

### Observations on vultures

For all birds and all phases, body mass was measured on the day of treatment (day 0) and at the end of each trial period or when birds were returned to their normal aviaries. For Phase I, II, III, and V, birds were weighed 12, 8, 12, and 5 d after treatment, respectively. Birds from Phase IV.1, IV.2, and VI were weighed on day 7. Body mass was measured to the nearest 0.5 kg (South Africa and Namibia) and 0.1 kg (India). Observations for signs of toxicity and abnormal feeding behaviour were undertaken daily. In Phases I–III, blood (2.5 ml) was taken at 0 h (prior to dosing) and at 4, 12, 24, 48, 96, and 148 h after meloxicam treatment to quantify serum uric acid and albumin concentrations and CK and ALT activity. In Phase IV, blood (5 ml) was taken just prior to dosing and 48 h and 168 h afterwards to determine serum uric acid concentrations. Blood sampling for Phase V was undertaken 24 h before feeding or dosing by oral gavage, and at 48 h and 96 h after dosing or start of feeding.

### Blood collection from vultures

In Phase I, blood samples were taken by use of an indwelling catheter, placed in the jugular vein while the vulture was under anaesthesia. This procedure was considered to be unsatisfactory and was rapidly abandoned. Subsequently, blood samples in all phases of the study were collected by direct veno-puncture from the brachial or tarsal veins. A total of approximately 15 ml of blood (about 3% of estimated blood volume) was collected from each vulture over a 7-d period.

### Measurement of serum constituents

Blood samples were spun at 1200 rpm for 15 min in a refrigerated centrifuge (4 °C) to separate serum. Uric acid concentration was measured using ACE TM Uric Acid Reagent, albumin concentration using the NExT TM Albumin reagent, ALT activity using the Alfa Wasserman ALT, and CK using the Alfa Wasserman CK Reagent e ACE TM clinical chemistry system (Alfa Wassermann, West Caldwell, New Jersey, United States; Bayer Health Care, Toronto, Canada). The analyses were performed by means of the ACE TM and NExT TM Clinical Chemistry Systems (Alfa Wassermann, Bayer Health Care).

## Supporting Information

Protocol S1 Estimating Likely MLE of Meloxicam (31 KB DOC).

Protocol S2 Analysis of Phase V Data (21 KB DOC).

Table S1 Blood Serum Constituents' Summary Statistics (69 KB DOC).

## Abbreviations

ALT: alanine transferase
EMEA: European Agency for the Evaluation of Medicinal Products
HPLC: high-performance liquid chromatography
MLE: maximum level of exposure
NSAID: nonsteroidal anti-inflammatory drug
UPBRC: University of Pretoria Biomedical Research Centre
