# Supplementary material for: Removing the Threat of Diclofenac to Critically Endangered Asian Vultures
Source: PLoS Biol. 2006 Jan 31;4(3):e66. doi: 10.1371/journal.pbio.0040066 (PMC1351921; doi:10.1371/journal.pbio.0040066)
Supplement: Supplementary file 1 [file pbio.0040066.sd001.doc]

### Protocol S1

**Estimating likely maximum level of exposure of meloxicam**

The results of a residue depletion study of meloxicam in cattle (EMEA 1997) were used to estimate the likely maximum level of exposure (MLE) of meloxicam to vultures. In this study, 16 Hereford/Friesian calves were injected subcutaneously with ^14^C-meloxicam at 0.7 mg kg^-1^ body weight for each of five consecutive days (EMEA 1997). Four animals were sacrificed at each of the following intervals after the final dose; 8 hours, 2, 4 and 8 days. Radioactive residue concentrations were measured in the liver, kidney, muscle, renal fat, omental fat and at the injection site. Residues were highest in the liver 8 hours after dosing, where concentrations reached 8,540 μg equivalents kg^-1^ (8.54 mg kg^-1^). Concentrations in all tissues declined subsequently, and, after 8 days, residues could only be measured in the liver (EMEA 1997). Making the worst case assumption that 100% of the radioactivity detected is associated with parent meloxicam (at 8 hours this association was estimated as 85%; EMEA 1997), this indicates a maximum exposure of 8.54 mg meloxicam for a vulture eating a 1 kg meal of liver. We calculated the daily food requirements of *G. bengalensis* following Mundy et al. (1992), amplified by notes from S. Piper. Daily energy use in kJ d^-1^ by free-living vultures was taken to be = 668.4**W*^0.622^, where W is vulture body weight in kg. Assuming that the mean body weight of wild *G. bengalensis* is 4.75 kg (range 3.5-6.0kg; Hoyo et al. 1994), this gives an energy use of 1763 kJ d^-1^. If the energy content of ungulate tissue is 6000 kJ kg^-1^ and the proportion of ingested energy assimilated is 0.86 (Mundy et al. 1992), then the daily requirement of ungulate tissue is 1763/(6000*0.86) = 0.341 kg. From observations of Cape Griffon vultures (*Gyps coprotheres*) it is estimated that birds can eat a single meal capable of supporting their energetic requirements for about three days (S. Piper, personal communication). We therefore assumed that a vulture is unlikely to consume more than about 1.02 kg of ungulate tissue in a single meal. Should a 4.75kg *G. bengalensis* gorge itself on liver tissue from a dead calf that had died 8 hours after receiving the last dose from a 5-day course of meloxicam at 0.7 mg kg^-1^, then this would indicate a likely MLE to meloxicam of (8.54x1.02) / 4.75 = 1.83 mg kg^-1^ vulture body weight. It is unlikely that the coincidence of conditions assumed above would occur frequently in the wild, thus we consider that 1.83 mg meloxicam/kg vulture body weight is the likely MLE in the wild. As a result, dose rates of meloxicam for safety testing were selected to cover a range up to 2 mg kg^-1^.

## References

EMEA (1997) The European Agency for the Evaluation of Medicinal Products. Committee for Veterinary Medicinal Products, Meloxicam Summary Report (1) EMEA/MRL/236/97-FINAL June 1997.

Del Hoyo J, Elliott A, Sartagal J, (1994) Handbook of the Birds of the World, Volume 2: New World Vultures to Guineafowl. Lynx Edicions. 638p.

Mundy PJ, Butchart D, Ledger J, Piper S. (1992) The vultures of Africa. Acorn, Johannesburg. 460p.
