# Supplementary material for: Removing the Threat of Diclofenac to Critically Endangered Asian Vultures
Source: PLoS Biol. 2006 Jan 31;4(3):e66. doi: 10.1371/journal.pbio.0040066 (PMC1351921; doi:10.1371/journal.pbio.0040066)
Supplement: Supplementary file 2 [file pbio.0040066.sd002.doc]

### Protocol S2

Analysis of Phase V data

In addition to the regression analyses reported in the main text, we also examined the Phase V data for subtle effects of feeding *G. africanus* with liver or muscle from meloxicam-treated cattle and dosing with meloxicam by gavage. We carried out analyses of covariance with the log of serum uric acid concentration as the dependent variable. We fitted regression models by ordinary least squares. Independent variables included in the full model were bird identity (BIRD a 6-level factor: 6 birds), stage of the experimental sequence (SEQ a 3-level factor: first, second or third set of trials), time of blood sampling (T a 2-level factor: 48 h or 96 h after treatment began), route by which meloxicam was administered (TREAT a 3-level factor: by feeding liver, by feeding muscle, by gavage), log dose of meloxicam administered (LDOSE a continuous variable) and the log uric acid concentration in the serum each bird before meloxicam was administered in a given trial (LURP a continuous variable). We tested the effect of meloxicam treatment by comparing the following models: (A) BIRD + SEQ + T + LURP + TREAT + LDOSE + TREAT.LDOSE and (B) BIRD + SEQ + T + LURP. This comparison indicated that there was no significant effect of the route of meloxicam dosing and meloxicam dose on serum uric acid level (*F_5,21_* = 0.58, *p* > 0.50). This was also the case when the equivalent analysis was performed separately for data collected 48 h after dosing and 96 h after dosing and when the tissue feeding and gavage routes of administration were subject to two separate analyses. We also fitted models to the full dataset with all possible combinations of the variables listed above, including the TREAT.LDOSE interaction. We then used the results to select the Minimal Adequate Model (MAM; Crawley 1993). The MAM was BIRD + LURP. In this model, both of these two variables had highly significant effects (BIRD: *F_5,29_* = 5.34, *p* < 0.005; LURP: *F_1,29_* = 17.26, *p* < 0.001), but no other variable or combination of variables had a significant further effect (*p* > 0.10) when added to the MAM.

### Reference

Crawley, M.J. (1993) GLIM for Ecologists. Blackwell Scientific Publications, Oxford. 379p.
