# Supplementary material for: Removing the Threat of Diclofenac to Critically Endangered Asian Vultures
Source: PLoS Biol. 2006 Jan 31;4(3):e66. doi: 10.1371/journal.pbio.0040066 (PMC1351921; doi:10.1371/journal.pbio.0040066)
Supplement: Supplementary file 3 [file pbio.0040066.st001.doc]

## Table S1. Blood serum constituents summary statistics

Results of analyses of measurements of uric acid and albumin concentrations, and alanine transferase (ALT) and creatinine kinase (CK) activity in blood serum before dosing and at 0, 4, 12, 23, 48, 96 and 168 hours after dosing for birds from Phase I (meloxicam at 0.5 mg kg^-1^), Phase II (1.0 mg kg^-1^) and Phase III and Phase IV (2.0 mg kg^-1^). Blood was only collected at 0, 48 and 168 hours in Phase IV. The geometric mean of meloxicam-dosed and control (sham-treated) birds is presented along with the sample size and *P* values from two-tailed t-tests between meloxicam-dosed and control groups.

|  |  | Uric acid mg l^-1^ | | | Albumin mg l^-1^ | | | ALT U l^-1^ (37° C) | | | CK U l^-1^ (37° C) | | |
| --- | --- | --- | --- | --- | --- | --- | --- | --- | --- | --- | --- | --- | --- |
|  |  | Control | Dosed |  | Control | Dosed |  | Control | Dosed |  | Control | Dosed |  |
| Phase | Time h | Mean (n) | Mean (n) | *p* | Mean (n) | Mean (n) | *p* | Mean (n) | Mean (n) | *p* | Mean (n) | Mean (n) | *p* |
| Phase I dose 0.5 mg kg^-1^ | 0 | 34.5 (3) | 40.2 (5) | > 0.80 | 11.3 (3) | 13.1 (5) | > 0.10 | 21.0 (3) | 33.1 (5) | > 0.10 | 432.2 (3) | 235.1 (5) | > 0.05 |
|  | 4 | 23.9 (3) | 32.1 (5) | > 0.40 | 11.3 (3) | 13.8 (5) | > 0.20 | 45.7 (3) | 38.4 (5) | > 0.70 | 591.1 (3) | 459.7 (5) | > 0.30 |
|  | 12 | 116.9 (3) | 106.5 (5) | > 0.80 | 11.7 (3) | 13.3 (5) | > 0.30 | 31.9 (3) | 31.0 (5) | > 0.90 | 506.7 (3) | 472.6 (5) | > 0.80 |
|  | 24 | 44.7 (3) | 51.7 (5) | > 0.50 | 12.2 (3) | 14.2 (5) | > 0.20 | 43.0 (3) | 40.0 (5) | > 0.80 | 440.1 (3) | 443.5 (5) | > 0.95 |
|  | 48 | 48.7 (2) | 64.8 (4) | > 0.30 | 13.1 (2) | 15.2 (4) | > 0.20 | 69.8 (2) | 39.5 (4) | > 0.10 | 386.4 (2) | 347.1 (4) | > 0.80 |
|  | 96 | 55.6 (3) | 63.2 (5) | > 0.40 | 11.8 (3) | 13.9 (5) | > 0.05 | 51.3 (3) | 43.3 (5) | > 0.05 | 461.7 (3) | 603.2 (5) | > 0.30 |
|  | 168 | 99.8 (3) | 79.1 (5) | > 0.40 | 12.5 (3) | 14.0 (5) | > 0.10 | 51.3 (3) | 46.0 (5) | > 0.50 | 570.6 (3) | 539.0 (5) | > 0.80 |
| Phase II dose 1.0 mg kg^-1^ | 0 | 72.0 (3) | 80.2 (5) | > 0.70 | 11.1 (3) | 11.5 (5) | > 0.50 | 32.5 (3) | 19.0 (5) | > 0.20 | 178.0 (3) | 223.9 (5) | > 0.20 |
|  | 4 | 39.8 (3) | 32.0 (5) | > 0.20 | 12.1 (3) | 11.3 (5) | > 0.40 | 28.5 (3) | 23.1 (5) | > 0.50 | 442.2 (3) | 603.2 (5) | > 0.50 |
|  | 12 | 125.8 (3) | 150.4 (5) | > 0.60 | 17.7 (3) | 11.1 (5) | > 0.20 | 72.4 (3) | 37.5 (5) | > 0.30 | 842.4 (3) | 397.2 (5) | > 0.40 |
|  | 24 | 73.9 (3) | 55.6 (5) | > 0.30 | 10.1 (3) | 10.9 (5) | > 0.20 | 33.8 (3) | 44.4 (5) | > 0.20 | 327.8 (3) | 492.8 (5) | > 0.30 |
|  | 48 | 73.0 (3) | 93.5 (5) | > 0.50 | 10.4 (3) | 11.2 (5) | > 0.10 | 43.0 (3) | 44.9 (5) | > 0.80 | 278.7 (3) | 405.2 (5) | > 0.30 |
|  | 96 | 124.6 (3) | 154.5 (5) | > 0.40 | 11.8 (3) | 12.9 (5) | > 0.20 | 39.8 (3) | 42.5 (5) | > 0.70 | 316.4 (3) | 234.6 (5) | > 0.30 |
|  | 168 | 114.5 (3) | 116.3 (5) | > 0.95 | 10.6 (3) | 11.6 (5) | > 0.05 | 54.9 (3) | 50.8 (5) | > 0.60 | 327.1 (3) | 327.2 (5) | > 0.95 |
| Phase III dose 2.0 mg kg^-1^ | 0 | 60.1 (3) | 56.8 (5) | > 0.80 | 12.6 (3) | 11.6 (5) | > 0.05 | 76.6 (3) | 74.0 (5) | > 0.70 | 243.0 (3) | 318.1 (5) | > 0.40 |
|  | 4 | 21.2 (3) | 18.1 (5) | > 0.50 | 11.8 (3) | 10.6 (5) | > 0.10 | 37.2 (3) | 46.5 (5) | > 0.50 | 431.4 (3) | 459.3 (5) | > 0.70 |
|  | 12 | 96.3 (3) | 93.1 (5) | > 0.90 | 11.0 (3) | 10.0 (5) | > 0.10 | 36.6 (3) | 40.7 (5) | > 0.70 | 328.3 (3) | 506.9 (5) | > 0.05 |
|  | 24 | 63.8 (3) | 51.8 (5) | > 0.60 | 10.8 (3) | 10.1 (5) | > 0.40 | 40.6 (3) | 46.8 (5) | > 0.40 | 278.7 (3) | 438.9 (5) | > 0.10 |
|  | 48 | 65.4 (3) | 53.2 (5) | > 0.50 | 10.5 (3) | 9.7 (5) | > 0.30 | 47.9 (3) | 40.4 (5) | > 0.60 | 220.4 (3) | 357.6 (5) | > 0.10 |
|  | 96 | 76.5 (3) | 84.7 (5) | > 0.80 | 11.5 (3) | 10.2 (5) | > 0.10 | 55.2 (3) | 52.7 (5) | > 0.90 | 236.1 (3) | 429.3 (5) | > 0.20 |
|  | 168 | 73.3 (3) | 84.8 (5) | > 0.70 | 12.8 (3) | 11.7 (5) | > 0.20 | 44.3 (3) | 54.2 (5) | > 0.60 | 531.7 (3) | 647.2 (5) | > 0.60 |
| Phase IV dose 2.0 mg kg^-1^ | 0 | 95.4 (4) | 111.7 (21) | > 0.50 | - | - | - | - | - | - | - | - | - |
|  | 48 | 70.5 (4) | 102.9 (21) | > 0.10 | - | - | - | - | - | - | - | - | - |
|  | 168 | 100.2 (4) | 125.4 (21) | > 0.30 | - | - | - | - | - | - | - | - | - |
